# Engineering a pyridoxal 5’-phosphate supply for cadaverine production by using *Escherichia coli* whole-cell biocatalysis

**DOI:** 10.1038/srep15630

**Published:** 2015-10-22

**Authors:** Weichao Ma, Weijia Cao, Bowen Zhang, Kequan Chen, Quanzhen Liu, Yan Li, Pingkai Ouyang

**Affiliations:** 1 State Key Laboratory of Materials-Oriented Chemical Engineering, Nanjing Tech University, Nanjing 211816, P.R. China; 2 College of Biotechnology and Pharmaceutical Engineering, Nanjing Tech University, Nanjing 211816, P.R. China; 3 College of Bioengineering and Biotechnology, Tianshui Normal University, Tianshui 741001, P.R. China

## Abstract

Although the routes of *de novo* pyridoxal 5′-phosphate (PLP) biosynthesis have been well described, studies of the engineering of an intracellular PLP supply are limited, and the effects of cellular PLP levels on PLP-dependent enzyme-based whole-cell biocatalyst activity have not been described. To investigate the effects of PLP cofactor availability on whole-cell biocatalysis, the ribose 5-phosphate (R5P)-dependent pathway genes *pdxS* and *pdxT* of *Bacillus subtilis* were introduced into the lysine decarboxylase (CadA)-overexpressing *Escherichia coli* strain BL-CadA. This strain was then used as a whole-cell biocatalyst for cadaverine production from L-lysine. Co-expression strategies were evaluated, and the culture medium was optimised to improve the biocatalyst performance. As a result, the intracellular PLP concentration reached 1144 nmol/g_DCW_, and a specific cadaverine productivity of 25 g/g_DCW_/h was achieved; these values were 2.4-fold and 2.9-fold higher than those of unmodified BL-CadA, respectively. Additionally, the resulting strain AST3 showed a cadaverine titre (*p* = 0.143, α = 0.05) similar to that of the BL-CadA strain with the addition of 0.1 mM PLP. These approaches for improving intracellular PLP levels to enhance whole-cell lysine bioconversion activity show great promise for the engineering of a PLP cofactor to optimise whole-cell biocatalysis.

Pyridoxal 5′-phosphate (PLP), which is one of most versatile cofactors, is essential to over 160 enzymatic activities that are catalogued by the Enzyme Commission, corresponding to ~4% of all known cellular catalytic activities^1^,^2^. PLP-dependent enzymes serve vital roles in all living organisms and catalyze a number of diverse chemical reactions, such as decarboxylation, transamination, racemization, Cα–Cβ bond cleavage and α,β-elimination reactions^3^,^4^,^5^. The basic function of PLP in these transformations is to act as an “electron sink”, which stabilises negative charges generated at the α-carbon of the substrate during the respective reactions^3^,^6^. The detailed mechanisms of some PLP-dependant reactions have been extensively reviewed^7^. In addition, PLP-dependent enzymes play a critical role in human health because they participate in numerous processes, including the metabolism of neurotransmitters, one-carbon units, biogenic amines, tetrapyrrolic compounds, and amino sugars; the modulation of hormone function and transcription factors; and the regulation of immune function^3^,^8^,^9^. For this reason, a number of PLP-dependent enzymes are widely recognised drug targets^8^. Additionally, the proper function of the enzymes and thus optimal health are dependent upon adequate levels of PLP in the cell^10^,^11^.

In recent years, various PLP-dependent enzymes have been exploited in industrial applications, especially for whole-cell biocatalysis^5^,^6^. For instance, (R)-selective ω-transaminase saturated with PLP in whole *Escherichia coli* cells has been used for the synthesis of chiral amines from a non-natural ketone substrate^12^. In another example, *E. coli* whole cells overexpressing the phenylacetaldehyde synthase (PAAS) of *Rosa hybrida* cv. have been used to produce 2-phenylethanol from L-phe^13^. In our previous study, we generated the *E. coli* strain BL-DAB, a lysine decarboxylase-overexpressing whole-cell biocatalyst for cadaverine production from L-lysine. This strain is capable of producing 221 g/L cadaverine within 16 h, with a molar yield of 92%^14^. However, due to the low level of *in vivo* PLP^10^, external supplementation of costly PLP was required for the maintenance of proper function of PLP-dependent enzymes during whole-cell biocatalysis; consequently, the use of this strain would be impractical in large-scale production^15^,^16^. Thus, engineering a *de novo* biosynthetic pathway to increase cellular level of PLP would be a promising approach for the construction of an effective whole-cell biocatalyst.

There are two distinct *de novo* PLP synthesis pathways. The first is the DXP-dependent pathway, which is found in *E. coli* and a few members of the γ subdivision of proteobacteria. This pathway involves seven enzymes and utilises deoxyxylulose 5-phosphate (DXP) as a precursor^3^,^17^,^18^. The other *de novo* PLP synthesis pathway is the ribose 5-phosphate (R5P)-dependent pathway, which remarkably involves only two enzymes, PdxS (also referred to as Pdx1, SnzP, or YaaD) and PdxT (also referred to as Pdx2, SnoP, or YaaE), and is widely distributed among various groups, including fungi, plants, the majority of eubacteria and archaea^19^,^20^,^21^,^22^. PdxS and PdxT form a hetero-oligomeric complex that functions as a glutamine amidotransferase, which utilises ribose 5-phosphate (R5P), glyceraldehyde 3-phosphate (G3P) and glutamine to directly synthesise PLP^17^,^19^,^20^,^22^,^23^,^24^. Therefore, it is seems reasonable that the introduction of the R5P-dependent pathway into *E. coli* would result in the efficient accumulation of PLP *in vivo*.

Lysine decarboxylase is one of the PLP-fold type I enzymes and catalyzes the decarboxylation of lysine to cadaverine (also known as 1,5-diaminopentane), which is an important platform chemical used in the production of various bio-based polyamides^25^,^26^,^27^. These bio-based polyamides exhibit many attractive properties and could compete with the conventional petroleum-based polyamides in all examined fields^28^,^29^. Moreover, these polyamides are of special interest due to the increasing focus on a low-carbon bio-economy. With respect to the biological production of cadaverine, whole-cell bioconversion has been proven to be a promising method due to its high efficiency^30^ and the economic viability of the precursor L-lysine, of which almost 2 million tons is produced annually^27^,^31^. One of the key issues with respect to cadaverine production by whole cells is biocatalyst stability, i.e., the maintenance of lysine decarboxylase activity for long reaction times. Stability is an important issue because it strongly influences both volumetric productivity and final product yield. Lysine decarboxylase activity is responsive to PLP binding, and it has been reported that when purified lysine decarboxylase (8% residual activity) is reconstituted with excess cofactor PLP, more than 90% reactivation was achieved^32^. It has also been observed that the fermentative cadaverine yield increases by 50% with the addition of 1 mg/L PLP in medium^33^. However, no reports have described the effect of intracellular PLP on whole-cell biocatalyst activity, and there is no prior knowledge regarding the engineering of an *in vivo* supply of PLP to enhance cadaverine production.

In the present study, the R5P- dependent pathway genes *pdxS* and *pdxT* of *B. subtilis* were introduced into the *E. coli* strain BL-CadA, a lysine decarboxylase-overexpressing whole-cell biocatalyst. This strain was examined for cadaverine production from L-lysine, and the corresponding change in intracellular level of PLP was determined. Furthermore, the co-expression of lysine decarboxylase and PdxST was optimised, and the scale-up fed-batch bioconversion of L-lysine to cadaverine using the PLP accumulation strain was performed. The results indicated that the intracellular concentration of PLP in the resulting strain reached 1144 nmol per gram dry cell weight (DCW), and the highest achieved specific cadaverine productivity rate was 25 g cadaverine/g_DCW_/h. Our results provide useful information for the application of PLP cofactor engineering in the construction of whole-cell biocatalysts to produce platform chemicals.

## Results

### Effect of PLP supplementation on whole-cell biocatalyst activity

The effects of PLP addition on cadaverine yield and productivity were investigated using whole cells of strain BL-CadA *E. coli*. As shown in Table 1, when PLP was absent, the molar yield of cadaverine over lysine (Y_Cadaverine/Lysine_) was 0.94 mol/mol at 3 h; the yield then decreased to 0.62 mol/mol at 9 h. Meanwhile, cadaverine productivity decreased from 4.11 g/g_DCW_/h to 2.43 g/g_DCW_/h. In comparison, with the addition of 0.1 mM PLP, the yield of cadaverine remained above 0.90 mol/mol during the bioconversion process, and the specific cadaverine productivity decreased slightly from 4.10 g/g_DCW_/h at 3 h to 3.68 g/g_DCW_/h at 9 h. These results indicated that the addition of PLP led to a longer continuation of the reaction and thus a higher final cadaverine concentration (70.4 g/L and 104 g/L with 0 and 0.1 mM PLP, respectively). The enhanced whole-cell biocatalysis activity upon PLP supplementation implied that the lysine decarboxylase was not fully activated, owing to the inadequate supply of cofactor PLP. Thus, it was expected that whole-cell activity would be enhanced by increasing the *in vivo* PLP concentration.

### Enhancement of intracellular level of PLP by the overexpression of R5P-dependent pathway genes

**Figure 1:**
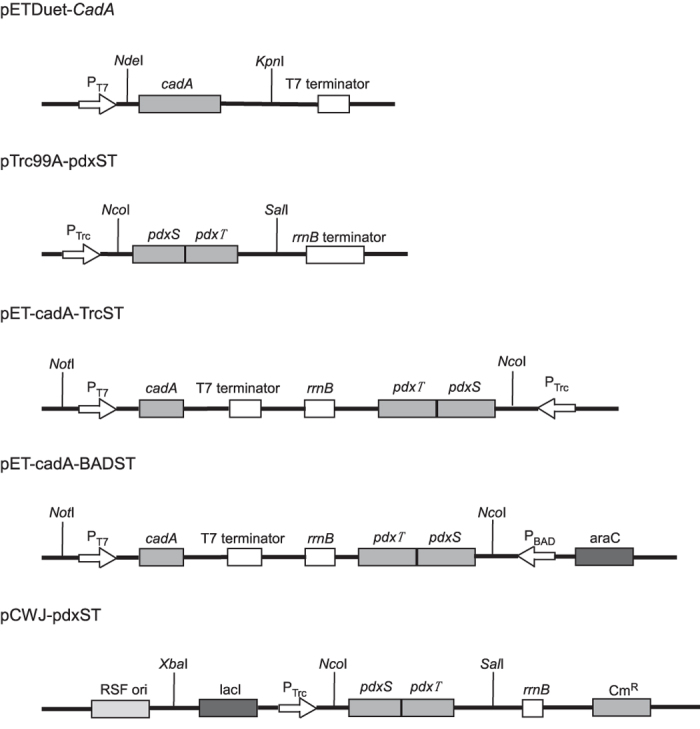
Schematic diagrams of the expression plasmids used in this study.

**Figure 2:**
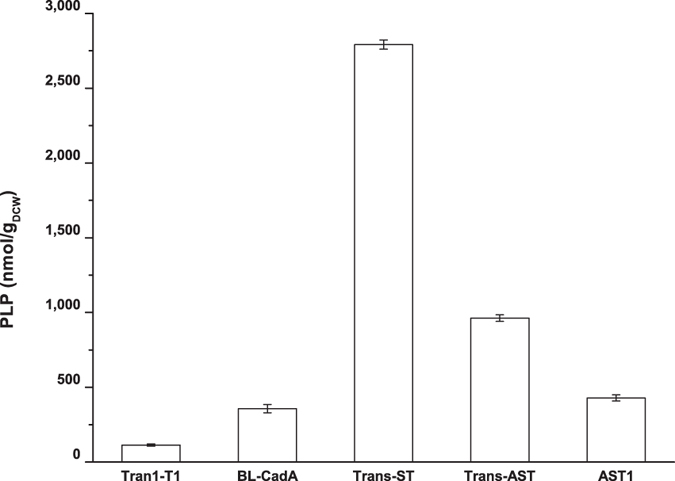
The concentration of intracellular PLP in the Trans1-T1, BL-CadA, Trans-ST, Trans-AST and AST1 strains. BL-CadA, *E. coli* BL21(DE3) harbouring the pETDuet-CadA plasmid (P_T7_-controlled *cadA*); Trans-ST, *E. coli* Trans1-T1 harbouring the pTrc99A-pdxST plasmid (P_trc_-controlled *pdxST*); Trans-AST, *E. coli* Trans1-T1 harbouring the pET-cadA-TrcST plasmid (P_T7_-controlled *cadA* and P_trc_-controlled *pdxST*); AST1, *E. coli* BL21(DE3) harbouring the pET-cadA-TrcST plasmid. The error bars indicate the standard deviation, as determined from triplicate experiments (three independent bacterial cultures).

To enhance the intracellular PLP supply, the *pdxST* operon of the R5P-dependent *de novo* PLP synthesis pathway was amplified from a *Bacillus subtilis* NJ308 Genomic DNA template and cloned into *Nco*I and *Sal*I sites of pTrc99A, resulting in pTrc99A-pdxST (Fig. 1). The amplified *pdxST* fragment was sequenced and submitted to GenBank (access No. KR821087). pTrc99A-pdxST was then introduced into *E. coli* strains, and the concentration of intracellular PLP was determined as described in the Methods. The results showed (Fig. 2) that the intracellular PLP level in the Trans-ST strain (Table S1) reached 2792 nmol/g_DCW_, which was 25-fold higher than that of Trans1-T1 (Table S1), which reached only 113 nmol/g_DCW_. This result indicated that the intracellular PLP level was improved by the expression of PdxS and PdxT. However, the PLP level in strain AST1 (Table S1) decreased to 429 nmol/g_DCW_, which was only slightly higher than the 357 nmol/g_DCW_ observed in the BL-CadA strain (Table S1). This decrease might be due to the low copy number of pET-cadA-TrcST (Fig. 1) or the influence of the co-expression of lysine decarboxylase. To address these possibilities, pET-cadA-TrcST was transformed into *E. coli* strain Trans1-T1 to yield strain Trans-AST (Table S1), which did not express the lysine decarboxylase, owing to the lack of T7 RNA polymerase. The concentration of intracellular PLP in resulting strain Trans-AST increased to 962 nmol/g_DCW_, which was 2.2-fold higher than that of AST1 but only 34% of that of Trans-ST. The results indicated that the expression of lysine decarboxylase and the plasmid copies both contribute to the low expression of proteins PdxST; these conclusions were further verified by SDS-PAGE analysis (supplementary Fig. S1).

### Co-expression of lysine decarboxylase and PdxST

**Figure 3:**
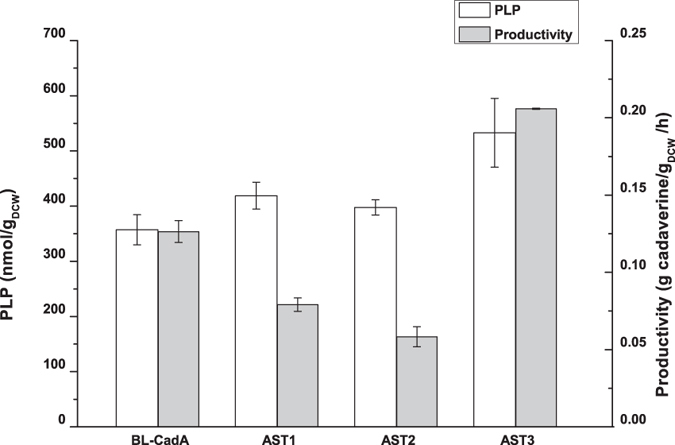
The concentration of intracellular PLP and cadaverine productivity of the BL-CadA, AST1, AST2, and AST3 strains. BL-CadA, *E. coli* BL21(DE3) harbouring the pETDuet-CadA plasmid (P_T7_-controlled *cadA*); AST1, *E. coli* BL21(DE3) harbouring the pET-cadA-TrcST plasmid (P_T7_-controlled *cadA* and P_trc_-controlled *pdxST*); AST2, *E. coli* BL21(DE3) harbouring the pET-cadA-BADST plasmid (P_BAD_-controlled *pdxST* and P_T7_-controlled *cadA*); AST3, *E. coli* BL21(DE3) harbouring the pETDuet-CadA and pCWJ-pdxST plasmids (P_trc_-controlled *pdxST*). The samples were collected after 6 h of induction with IPTG. The error bars indicate the standard deviation, as determined from triplicate experiments (three independent bacterial cultures).

To optimise the co-expression of proteins in the whole-cell biocatalyst, two other strains with different co-expression methods were constructed. In strain AST2, lysine decarboxylase and PdxST were expressed from the same vector used in AST1, except that the *pdxST* genes were placed under the control of the arabinose-inducible araBAD promoter (Fig. 1). In strain AST3, two compatible vectors were used for the expression of lysine decarboxylase and PdxST, respectively. As shown in Fig. 3, a significant decrease (*p* < 0.001, α = 0.05) in cadaverine productivity was observed in strains AST1 and AST2, in which the proteins were transcribed from single vector; however, the intracellular PLP level increased slightly. The reason for the decrease in biocatalyst activity was the poor expression of lysine decarboxylase, which was affected by the co-expression of PdxST, as shown in Supplementary Fig. S1. In comparison, a 1.7-fold increase in cadaverine productivity and a 1.5-fold increase in cellular PLP level were observed in AST3 over the BL-CadA control. These results suggested that the transcription of respective genes from two vectors was better for protein co-expression, a result consistent with those from a previous report showing many examples of improved expression with the use of multi-vectors compared with one-vector strategies^34^. These results also demonstrated that the higher cadaverine productivity resulted from a higher level of *in vivo* PLP, provided that lysine decarboxylase was effectively expressed. Although the concentration of PLP in AST3 reached 533 nmol/g_DCW_, this value was only 5.33 μM PLP in the biotransformation medium when 10 g_DCW_/L of whole-cell biocatalyst used. Thus, because the activity of lysine decarboxylase was proportional to the amount of cofactor present^32^, it was still necessary to improve the level of cellular PLP. Moreover, comparison with the intracellular PLP level the Trans-ST strain (2792 nmol/g_DCW_) suggested further room for improvement. Because the level of protein expression and PLP biosynthesis could differ significantly under varied culture conditions, leading to drastic performance differences for whole-cell biocatalysis, the culture medium was optimised^35^.

### Optimization of the culture medium for a balanced expression of lysine decarboxylase and PdxST

**Figure 4:**
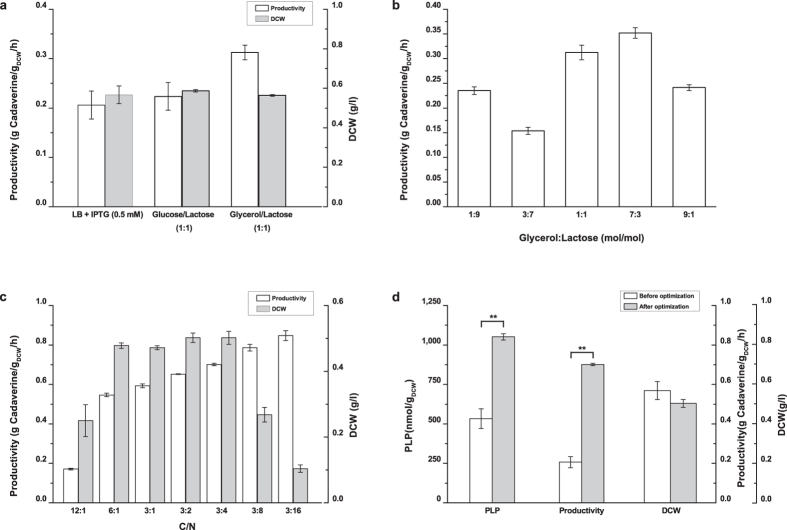
The effect of culture medium composition on cell growth, cadaverine production and intracellular PLP levels in strain AST3. AST3, *E. coli* BL21(DE3) harbouring pETDuet-CadA and pCWJ-pdxST. (**a**) Comparison of cell growth and cadaverine productivity of AST3 cultured in medium with different carbon sources. (**b**) Cadaverine productivity of whole-cell AST3 cultured in medium with different glycerol:lactose ratios. (**c**) Comparison of cell growth and the cadaverine productivity of AST3 cultured in medium with different C/N ratios. (**d**) Effect of culture medium optimization on intracellular PLP level, cadaverine production and cell growth of strain AST3. The samples were collected after 6 h of induction with IPTG or 12 h of incubation with lactose. The error bars indicate the standard deviation, as determined from triplicate experiments (three independent bacterial cultures). **Indicates significant difference (*p* < 0.01, α = 0.05). For clarity, not all significant differences are shown.

Glycerol is the precursor of glyceraldehyde 3-phosphate (G3P), which is the substrate for pyridoxal biosynthesis lyase (PdxS) in the synthesis of PLP^24^,^36^. Thus, the possibility of using glycerol instead of glucose as the carbon source was investigated. Furthermore, lactose was used for the induction of protein expression to substitute for isopropyl β-D-1-thiogalactopyranoside (IPTG), which is impractical in large-scale production^37^. As shown in Fig. 4a, a significant (*p* = 0.004, α = 0.05) increase in cadaverine productivity was observed, and the highest specific productivity, 0.31 g cadaverine/g_DCW_/h, was obtained by cells cultivated in glycerol-lactose medium, which was 1.5 and 1.4-fold higher than that in LB medium (IPTG induced) and glucose-lactose medium, respectively. Moreover, no significant differences in microbial biomass were observed among the cultures. The results indicated that glycerol can be used as carbon source and has a positive effect on whole-cell biocatalyst activity without affecting cell growth.

The effects of the glycerol:lactose and C/N ratios in the culture medium on biocatalyst activity were further investigated. The results showed that cadaverine productivity increased significantly (*p* < 0.001, α = 0.05) when the ratio of glycerol to lactose in the culture medium was 7:3 (mol/mol) (Fig. 4b). Productivity was further increased with a decrease in the C/N ratio in the medium (Fig. 4c), whereas the harvested microbial biomass was significantly (*p* < 0.001, α = 0.05) decreased when the C/N ratio of medium was lower than 3:8. These results suggested that a glycerol to lactose ratio of 7:3 and a C/N ratio of 3:4 in culture medium were optimal for the cultivation of whole-cell biocatalysts for cadaverine production. Under these cultivation conditions, a specific productivity of 0.70 g cadaverine/g_DCW_/h and an intracellular PLP concentration of 1051 nmol/g_DCW_ were achieved; these values were 3.5-fold and 2.0-fold higher, respectively, compared with those obtained before optimization (Fig. 4d). In addition, no significant decrease (*p* = 0.163, α = 0.05) in microbial biomass was observed when growing cells in the optimised medium (Fig. 4d).

### Scale-Up of the Fed-Batch Bioconversion

**Figure 5:**
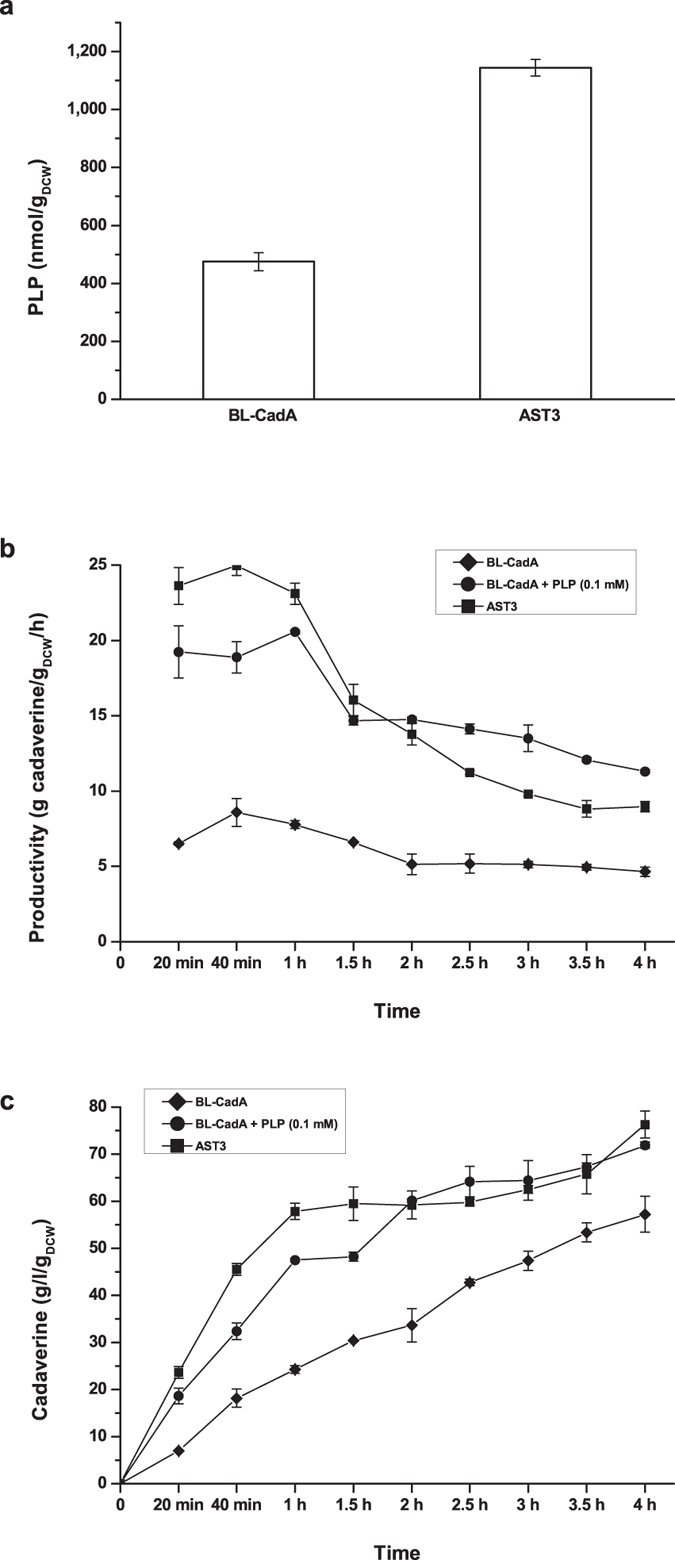
Fed-batch bioconversion for cadaverine production. (**a**) The intracellular PLP concentration in the BL-CadA and AST3 strains. The samples were collected after 6 h of induction with IPTG or 12 h of incubation with lactose. (**b**) The cadaverine productivity of strain AST3 (■), BL-CadA without PLP supplementation (♦), and BL-CadA with the addition of 0.1 mM PLP (•). (**c**) The cadaverine yield of the strains. The error bars indicate the standard deviation, as determined from triplicate experiments (three independent bacterial cultures).

To validate the suitability of the whole-cell biocatalyst for cadaverine production, the BL-CadA and AST3 strains were cultivated in a modified M9 medium with a glycerol to lactose ratio of 7:3 and a C/N ratio of 3:4 for 12 h under the conditions described in the Methods. The scale-up tests of fed-batch bioconversion were carried out in a 1-L fermenter (Infors, Switzerland) with an initial volume of 0.3 L of biotransformation mixture containing approximately 10 g_DCW_/L of whole-cell biocatalyst. The results showed that the intracellular concentration of PLP (Fig. 5a) in strain AST3 reached 1144 nmol/g_DCW_, which was 2.4-fold higher than that of BL-CadA (475 nmol/g_DCW_). The highest specific cadaverine productivity of strain AST3 reached 25 g cadaverine/g_DCW_/h, which was 1.2-fold and 2.9-fold higher than that of BL-CadA with and without PLP supplementation, respectively. In the first hour of bioconversion, the specific cadaverine productivity rate of the AST3 strain remained at 24 ± 1 g cadaverine/g_DCW_/h, which was 3 times higher than that of BL-CadA (8 ± 1 g cadaverine/g_DCW_/h) and 1.2-fold higher than the PLP supplementation group (20 ± 1 g cadaverine/g_DCW_/these) (Fig. 5b). After 4 hours of bioconversion, the AST3 strain produced 76 g/L cadaverine per gram biocatalyst (Fig. 5c), which was 1.3-fold higher than BL-CadA. Additionally, AST3 showed a similar cadaverine titre (*p* = 0.143, α = 0.05) as that in BL-CadA with the addition of 0.1 mM PLP.

## Discussion

The critical role of *in vivo* PLP in reactions catalyzed by PLP-dependent enzymes has been studied extensively^1^,^24^. However, limited studies have examined the effect of intracellular PLP levels on stimulating PLP-enzyme activity in a whole-cell biocatalyst. In this study, we found that the addition of PLP led to an extended continuation of the lysine decarboxylation reaction (Table 1), which is consistent with a previously reported observation^13^,^15^,^16^,^38^. The requirement for external supplementation of PLP in a whole-cell bioconversion process indicated that the free PLP inside the cell was insufficient to maintain enzyme activity. Thus, it was expected that the increase in cellular PLP level by the enhancement of *de novo* synthesis should have effects on whole-cell biocatalysis. To increase the *de novo* PLP synthesis, PdxS and PdxT from *Bacillus subtilis* were over-expressed in *E. coli* in this study. These are proteins of the R5P-dependent PLP biosynthesis pathway and resulted in a 25-fold increase (from 113 nmol/g_DCW_ to 2792 nmol/g_DCW_) in the observed PLP intracellular levels (Fig. 2). These results confirmed that PLP can accumulate *in vivo* to a high level. It was also found that whole-cell biocatalysts efficiency was positively correlated with the cellular PLP concentration, as shown in the AST3 strain (Fig. 3).

If the enzymes of the *de novo* pathway of PLP synthesis are effectively expressed, the culture medium can also affect the cellular level of PLP and thus biocatalyst efficiency. Therefore, the composition of culture medium was further optimised by using a glycerol-lactose medium. Glycerol is a commonly used carbon source for recombinant protein expression in *E. coli* cells because low acetate levels and higher cAMP concentrations have been detected in glycerol-grown cells^39^,^40^. As previously documented, recombinant protein expression is decreased by acetate accumulation^39^,^41^ and stimulated by the CAP/cAMP complex^42^. In addition, before glycerol can enter the glycolysis or gluconeogenesis pathways, it must first be converted to the intermediate glyceraldehyde 3-phosphate, which is one of the PLP-building blocks. Thus, the effects of using glycerol as a carbon source on the intracellular PLP supply and whole-cell activity were investigated in this study.

Our results showed that the biocatalyst activity for cadaverine production was significantly increased when glycerol was used as carbon source (Fig. 4a). Specifically, a 1.75-fold increase in cadaverine productivity was obtained by using the biocatalyst harvested in culture medium containing a glycerol to lactose ratio of 7:3 (Fig. 4b). We also found that the biocatalyst activity was augmented with increasing ammonium concentrations in the culture medium (Fig. 4c). This result is consistent with those from previous reports on the ammonium-dependent PLP synthesis activity of PdxS^43^. However, a decrease in microbial biomass was observed when the C/N ratio of the medium was lower than 3:8 (ammonium chloride 8 g/L). This observation may be closely related to the inhibition effect of high ammonium concentrations on cell growth^44^. Thus, a compromise was made wherein a C/N ratio of 3:4 in the culture medium was used for cultivation of the whole-cell biocatalyst. Medium optimization, improved the cellular PLP concentration and cadaverine productivity by approximately 2.0-fold and 3.5-fold, respectively, compared with the values obtained for AST3 cultured in the original LB medium. Additionally, no significant changes (*p* = 0.163, α = 0.05) in microbial biomass were observed (Fig. 4d). The results that were obtained in the fed-batch bioconversion showed that the cadaverine productivity of strain AST3 was 1.2-fold higher than that of the control strain BL-CadA with the addition of 0.1 mM PLP; in these conditions, a specific cadaverine productivity of 25 g cadaverine/g_DCW_/h was achieved. These results implied that the performance of the whole-cell biocatalyst was effectively improved upon the increase in cellular PLP level, and additional PLP supplementation was not required for cadaverine production.

Although much work has been performed in the field of PLP-dependent enzymes, the effects and corresponding mechanisms of the availability of cellular PLP in sustaining PLP-enzyme activity remain to be fully elucidated. One reason for this lack of understanding is that the effect of PLP appears to vary among PLP-dependent enzymes. In some instances, such as for *E. coli* serine hydroxymethyltransferase^45^,^46^ and MalY^47^, PLP binds at the end of the folding pathway and plays a significant role in stabilizing the native dimeric apoenzyme. In contrast, the β_2_ subunit of tryptophan synthase from *E. coli*^48^ and *Treponema denticola* cystalysin^49^ require PLP for refolding *in vitro*, suggesting that PLP could improve folding efficiency. In addition, a study of human alanine:glyoxylate aminotransferase (AGT) has shown that the catalytic activity of AGT in stably transformed CHO cells is highly dependent on the intracellular concentrations of its cofactor PLP. In this context, PLP aids in AGT’s folding and/or dimerisation and thereby its acquisition of catalytic activity^50^. With respect to lysine decarboxylase, which was addressed in this study, both the mechanism of PLP binding and the correlation between increased whole-cell biocatalyst activity and increased *in vivo* PLP concentrations are unclear. However, various *in vitro* and *in vivo* data indicate that the increased intracellular concentration of PLP likely exerts effects by (i) increasing the enzyme’s catalytic activity by shifting the equilibrium from the apo form to the more stable holo form and (ii) improving the folding efficiency and/or increasing the enzyme’s stability against intracellular degradation^11^,^47^,^50^.

In summary, using a lysine decarboxylase-overexpressing whole-cell biocatalyst as a model system, we enhanced the catalytic efficiency by increasing intracellular PLP levels, which was achieved by introducing the PdxS and PdxT proteins of *Bacillus subtilis*. To the best of our knowledge, this is the first report on the engineering of a PLP *de novo* synthesis pathway in *E. coli* to construct a whole-cell biocatalyst for cadaverine production without the external addition of PLP co-factor. Furthermore, the approaches developed in this study should be applicable to other whole-cell systems that are based on PLP-dependent enzymes.

## Methods

### Construction of strains and plasmids

The bacterial strains and plasmids used in this study are summarised in Supplementary Table S1. All of the PCR primers are listed in Supplementary Table S2. The DNA manipulations were performed according to standard protocols. The constructed plasmids were confirmed by enzymatic digestion and DNA sequencing, which was performed by Genewiz (Suzhou, China).

The *E. coli* lysine decarboxylase gene (*cadA*) was amplified from *E. coli* BL21 (DE3) genomic DNA (GenBank: AM946981.2) and introduced into pETDuet-1 to generate pETDuet-CadA (Fig. 1). The operon comprising *pdxS* and *pdxT* was amplified from *Bacillus subtilis* NJ308 Genomic DNA template and then ligated into pTrc99A to generate the pTrc99A-pdxST plasmid (Fig. 1). The *Ptrc* (*trc* promoter)-*pdxST*-rrnB cassette from pTrc99A-pdxST was joined with a linearised pETDuet-CadA to construct pET-cadA-TrcST (Fig. 1). The pKD46 fragment containing the *araBAD* promoter and the *araC* gene, together with the 4.2-kb fragment of pET-cadA-TrcST containing the T7 promoter-cadA and *pdxST* gene cassettes, were inserted into the *Not*I and *Avr*II sites of pETDuet-CadA to yield pET-cadA-BADST (Fig. 1). To construct pCWJ-pdxST (Fig. 1), the fragment containing the *pdxST* coding sequence was digested by *Nco*I and *Sal*I and ligated into the same restriction sites of plasmid pCWJ. The constructed plasmids pTrc99A-pdxST and pET-cadA-TrcST were separately transformed into *E. coli* Trans1-T1 (TransGen Biotech, Beijing, China) to create the Trans-ST and Trans-AST strains, respectively (Table S1). The pETDuet-CadA, pET-cadA-TrcST and pET-cadA-BADST plasmids were separately transformed into *E. coli* BL21(DE3) (TransGen Biotech, Beijing, China) to yield the BL-CadA, AST1 and AST2, strains, respectively (Table S1). The pETDuet-CadA and pCWJ-pdxST plasmids were co-transformed into *E. coli* BL21(DE3) and defined as strain AST3 (Table S1). The detailed protocols for plasmid and stain constructions are described in the Supplementary Materials.

### Whole-cell biocatalyst preparation

Recombinant *E. coli* cells harbouring the appropriate plasmid were each seeded into 50 mL of LB medium with appropriate antibiotics in 250-mL shaking flasks at an inoculation volume of 5%. Upon reaching an OD_600_ of 0.5, the cells were induced by 0.5 mM IPTG (or 10 mM L-arabinose if needed). After 6 h of incubation with shaking at 37 °C, the cells were harvested by centrifugation at 4,000 × g for 10 min at 4 °C and washed twice with 0.9% saline solution. For medium optimization, a modified M9 medium was used instead of LB medium. The modified M9 medium contained the following components (g/L): carbon source, 2; Na_2_HPO_4_•12H_2_O, 17.1; KH_2_PO_4_, 3; NH_4_Cl, 1 (or as specified); NaCl, 0.5; MgSO_4_, 0.12; CaCl_2_, 0.011; and 1 ml/L trace metals, as described elsewhere^51^. Large-scale cultivation was carried out using the semi-defined medium (glycerol 1.15 ml/L, lactose 0.6 g/L, NH_4_Cl 4 g/L, Na_2_HPO_4_•12H_2_O 17.1 g/L, KH_2_PO_4_ 3 g/L, NaCl 0.5 g/L, yeast extract 0.05 g/L, peptone 0.1 g/L, MgSO_4_, 0.12 g/L; CaCl_2_, 11.1 mg/L; and trace metals 1 ml/L). A total of 400 mL of cultured cells were inoculated into a 7.5-L jar fermentor (BioFlo 115, New Brunswick Scientific Co., USA) containing 3.6 L of medium at an initial OD_600_ of ~0.2. The temperature was maintained at 37 °C, and the pH was adjusted at 7.0 by automatically adding 2 M NaOH. The dissolved oxygen level was maintained above 20% by supplying air at 1 vvm (air volume/working volume/min) and by automatically controlling the agitation speed up to 500 rpm. After 12 h of incubation, the cells were harvested by centrifugation at 4,000 × g for 10 min at 4 °C and washed twice with a 0.9% saline solution.

### Whole-cell biotransformation

To investigate the effects of PLP supplementation on whole-cell biocatalyst activity, the whole-cell biotransformations were conducted in a 125-mL serum bottle at 37 °C on a rotary shaker at 200 rpm, with a total biotransformation medium volume of 50 mL. The *E. coli* strain BL-CadA was cultured and induced as described above. The reaction mixture contained 100 g/L L-lysine monohydrochloride and 4 g_DCW_/L of whole-cell biocatalyst in a 0.9% saline solution. The volume of the reaction mixture and the concentrations of L-lysine and cadaverine in reaction mixture were determined every 3 hours. For the evaluation of protein expression and culture medium optimization, small-scale whole-cell biotransformations were conducted in a 50-mL centrifuge tube at 37 °C on a rotary shaker at 250 rpm, with a total biotransformation medium volume of 5 mL. The reaction mixture contained 12.5 g/L L-lysine monohydrochloride and 2 g_DCW_/L of whole-cell biocatalyst in a 0.9% saline solution. After 30 min incubation, the mixture was centrifuged at 6,000 × g for 5 min, and the cadaverine concentration in the supernatant was measured. The scale-up tests of fed-batch bioconversion were carried out in a 1-L fermentor (Infors, Switzerland) with an initial volume of 0.3 L of biotransformation mixture. This mixture contained 200 g/L L-lysine monohydrochloride and approximately 10 g_DCW_/L of whole-cell biocatalyst in a 0.9% saline solution. Biotransformation was conducted at 37 °C and 300 rpm to prevent cell sedimentation. The volume of reaction mixture and the concentration of L-lysine in the reaction mixture were determined periodically, and when the L-lysine concentration decreased to 20–30 g/L, L-lysine monohydrochloride powder was fed to a final concentration of 200 g/L.

### Analytical methods

The L-Lysine concentrations were determined by an SBA-40E immobilised enzyme biosensor (Shandong, China). The cadaverine and the intracellular PLP concentrations were determined by reverse-phase high-performance liquid chromatography (HPLC) using an Agilent (Santa Clara, CA, USA) 1290 Infinity System equipped with a fluorescence detector (FLD G1321B). The cadaverine concentration was determined by pre-column dansyl chloride derivatization following a previously described procedure^14^. The intracellular PLP level was determined following the procedure described by Cabo *et al.* and Kimura *et al.*, with modifications^52^,^53^. The specific steps are described in the Supplementary Methods.

### Statistical analysis

Experiments were conducted in triplicate. The values shown represent mean ± standard deviations (SD). The experimental data were analysed for variance by analysis of variance (ANOVA), followed by Tukey’s post-hoc analysis; mean differences were established by a two-tailed *t*-test at α =  0.05; *p* < 0.05 represented significant differences.

## Additional Information

**How to cite this article**: Ma, W. *et al.* Engineering a pyridoxal 5'-phosphate supply for cadaverine production by using *Escherichia coli* whole-cell biocatalysis. *Sci. Rep.* **5**, 15630; doi: 10.1038/srep15630 (2015).

## Supplementary Material

Supplementary Information

## Figures and Tables

**Table 1:** Summary of cadaverine production by whole-cell bioconversion with or without the addition of PLP*.

Conc. ofPLP (mM)	Reactiontime (h)	Conc. ofCadaverine(g/L)	Y_Cadaverine/Lysine_(mol/mol)	SpecificProductivity(g/g_DCW_/h)
0	3	58.0 ± 1.4	0.94 ± 0.03	4.11 ± 0.11
	6	65.9 ± 3.0	0.71 ± 0.04	2.89 ± 0.15
	9	70.4 ± 2.2	0.62 ± 0.02	2.43 ± 0.08
0.1	3	57.8 ± 0.1	0.96 ± 0.00	4.10 ± 0.00
	6	77.0 ± 0.4	0.90 ± 0.01	3.42 ± 0.02
	9	104 ± 1	0.95 ± 0.01	3.68 ± 0.03

^*^Each value is the mean ± standard deviation of three biological replicates (three independent bacterial cultures).
