# Supplementary material for: Engineering a pyridoxal 5’-phosphate supply for cadaverine production by using Escherichia coli whole-cell biocatalysis
Source: Sci Rep. 2015 Oct 22;5:15630. doi: 10.1038/srep15630 (PMC4614675; doi:10.1038/srep15630)
Supplement: Supplementary Information [file srep15630-s1.pdf]

SUPPLEMENTARY MATERIAL

Engineering a pyridoxal 5'-phosphate supply for cadaverine production by using *Escherichia coli* whole-cell biocatalysis

Weichao Ma[1,2,3], Weijia Cao[1,2], Bowen Zhang[1,2], Kequan Chen[1,2*], Quanzhen Liu[1,2], Yan Li[1,2], and Pingkai Ouyang[1,2]

Supplementary Methods

Construction of strains and plasmids

Bacterial strains and plasmids are summarized in Table S1. All the PCR primers are listed in Table S2.

The *E. coli cadA* gene, encoding a lysine decarboxylase protein, were amplified from *E. coli* BL21 (DE3) genomic DNA (GenBank: AM946981.2) using primers CadA-NdeI-Duet-F and CadA-KpnI-Duet-R. The PCR product was introduced into the *Nde*I and *Kpn*I sites of pETDuet-1 to generate pETDuet-CadA.

The operon comprising *pdxS* and *pdxT* was amplified from *Bacillus subtilis* NJ308 Genomic DNA template with primers pair PdxST-NcoI-F and PdxST-SalI-R, the PCR fragment was digested with *Nco*I and *Sal*I, and ligated into pTrc99A, generating pTrc99A-pdxST.

To construct pET-cadA-TrcST, the P*trc* (*trc* promoter)- *pdxST*-rrnB cassette was amplified with primers pair Trc-promoter-XbaI and Trc-termi-XbaI using pTrc99A-pdxST as the template. Then the fragment was digested by *Xba*I, and ligated into pETDuet-CadA linearized with *Avr*II.

The fragment containing the *araBAD* promoter and *araC* gene was amplified with the primers

Para-Spe-Xba-F and Para-Nco-Bam-R using plasmid pKD46 as the template and was subsequently digested with *Nco*I and *Xba*I. The resulting fragment, together with the 4.2-kb *Not*I-*Nco*I fragment of pET-cadA-TrcST containing the cassette of T7 promoter-cadA and *pdxST* genes, were inserted into the *Not*I and *Avr*II sites of pETDuet-CadA to yield pET-cadA-BADST.

To construct pCWJ-pdxST, the fragment containing *pdxST* coding sequence was digested by *Nco*I and *Sal*I, and ligated into the same restriction sites of plasmid pCWJ.

The constructed plasmids pTrc99A-pdxST and pET-cadA-TrcST were separately transformed into *E.coli* Trans1-T1 (TransGen Biotech, Beijing, China) to create strains Trans-ST and Trans-AST, respectively. The plasmids pETDuet-CadA, pET-cadA-TrcST and pET-cadA-BADST were separately transformed into *E.coli* BL21(DE3) (TransGen Biotech, Beijing, China) to yield strains BL-CadA, AST1 and AST2, respectively. The plasmids pETDuet-CadA and pCWJ-pdxST were co-transformed into *E.coli* BL21(DE3) and defined as strain AST3.

Determination of intracellular level of Pyridoxal 5'-phosphate

10 mg (dry cell weight) cell pellets of the 6 h IPTG-induced *E. coli* cells were resuspended in 1.5 mL phosphate buffered saline (PBS, pH 7.4, Sigma) containing 100 μg/mL lysozyme, 10 μg/mL RNase A , and 5 μg/mL DNase I. After incubation on ice for 1 hour, bacteria were broken by sonication, and 20 μg of Proteinase K was added to each sample and incubated on ice for 30 minutes. Then proteins were precipitated by adding chilled 100% Trichloracetic acid (TCA) at 1/10 of the sample volume. The samples were vortexed for 1 min and incubated 15 min on ice, and then were centrifuged for 10 min at 12,000×g at 4 °C. The supernatant was cleared by 0.2 μm filtration.

The separation, identification, and quantification of pyridoxal 5'-phosphate was conducted by

high-performance liquid chromatography (HPLC), following modifications of the procedure described by Cabo *et al.* and Kimura *et al.*[1,2], using an Agilent (Santa Clara, CA, USA) 1290 Infinity System equipped with a fluorescence detector (FLD G1321B). The stationary phase was a reverse-phase column Prevail C18 (250 mm × 4.6 mm × 5 μm) (Grace, Columbia, MD, USA). The mobile phase consisted of buffer A: 0.1 M potassium phosphate monobasic, 0.1 M sodium perchlorate and 0.5 g/L sodium bisulfite; buffer B: 0.1 M potassium phosphate monobasic, 0.1 M sodium perchlorate, 0.5 g/L sodium bisulfite and 20% acetonitrile. The pH of the buffers A and B were adjusted to 3.0 by adding phosphoric acid. The following gradient for the mobile phase was used: buffer A, 100%, from 0 to 4 min and buffer B, 27%, from 4.1 to 13 min. An additional 5-min step was included to reach the initial conditions and achieve mobile phase stabilization. The flow rate was 1 mL/min, and the injection volume was 20 μL. The temperature of the column was 25 ± 1 °C, and fluorescence was measured at an excitation wavelength of 300 nm and an emission wavelength of 400 nm.

1. Cabo, R. *et al.* A simple high-performance liquid chromatography (HPLC) method for the measurement of pyridoxal-5-phosphate and 4-pyridoxic acid in human plasma. *Clinica chimica acta; international journal of clinical chemistry* **433**, 150-156 (2014).
2. Kimura, M., Kanehira, K. & Yokoi, K. Highly sensitive and simple liquid chromatographic determination in plasma of B6 vitamers, especially pyridoxal 5`-phosphate. *Journal of Chromatography* **722**, 295-301 (1996).

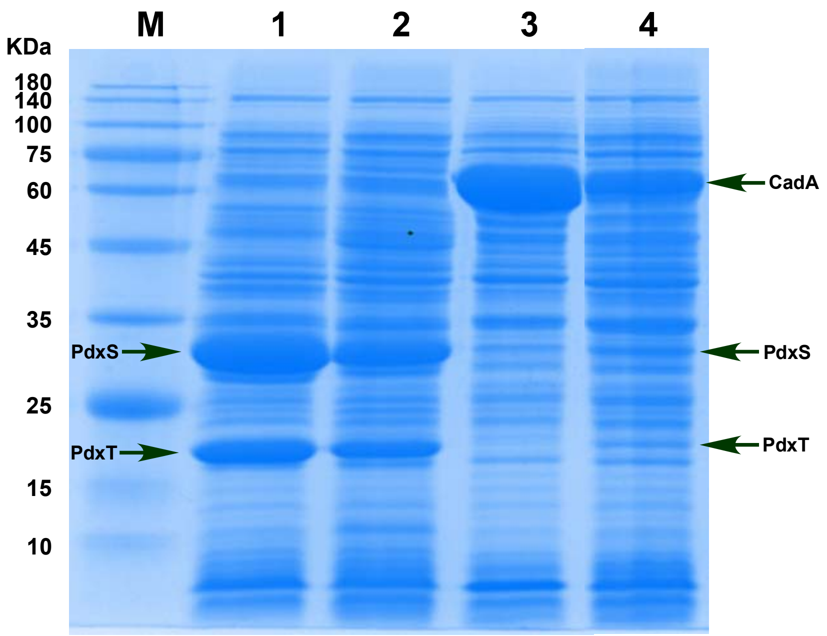

Supplementary Figure S1. SDS-PAGE analysis of the expression of PdxS, PdxT and CadA. Lane 1, total protein in strain Trans-ST; Lane 2, total protein in strain Trans-AST; Lane 3, total protein in strain BL-CadA; Lane 4, total protein in strain AST1. The samples were collected after 6 h of induction with IPTG.

Table S1. Strains and plasmids used in this work

Strains or plasmids	Description	Source or Refs.
Strains		
Bacillus subtilis NJ308	Wild-type	Lab stock
E.coli Trans1-T1	F^{-} φ80(*lac*Z)ΔM15 Δ*lac*X74 *hsd*R(r_K^-, m_K^+) Δ*rec*A1398 *end*A1 tonA; *E. coli* host for gene cloning and for expression of proteins under the control of the trc promoter	TransGen Biotech
E.coli BL21(DE3)	*F^{-} omp*T *hsd*S(r_B^-, m_B^-) *gal dcm* (DE3); *E. coli* host for protein expression	TransGen Biotech
BL-CadA	BL21(DE3) harboring pETDuet-CadA	Ma et al.
Trans-ST	*E.coli* Trans1-T1 harboring pTrc99A-pdxST	This work
Trans-AST	*E.coli* Trans1-T1 harboring pET-cadA-TrcST	This work
AST1	BL21(DE3) harboring pET-cadA-TrcST	This work
AST2	BL21(DE3) harboring pET-cadA-BADST	This work
AST3	BL21(DE3) harboring pETDuet-CadA and pCWJ-pdxST	This work
Plasmids		
pETDuet-1	ColE1 *ori*, *lac*I gene, Amp^r, T7 promoters	Novagen
pTrc99A	pMB1 *ori*, *lac*I gene, Amp^r, trc promoter	Lab stock
pCWJ	RSF *ori*, *lac*I gene, Cm^r, trc promoter	Lab stock
pKD46	pSC101 *ori*, Amp^r, λ Red recombinase under arabinose-inducible araBAD promoter	Lab stock
pETDuet-CadA	ColE1 *ori*, *lac*I gene, Amp^r, T7 promoters, *cadA* inserted between *Nde*I–*Kpn*I sites of pETDuet-1	Ma et al.
pTrc99A-pdxST	pMB1 *ori*, *lac*I gene, Amp^r, trc promoter, Native *pdxST* operon of *B. subtilis* NJ308 inserted between the *Nco*I and *Sal*I sites of pTrc99A	This work
pET-cadA-TrcST	ColE1 *ori*, *lac*I gene, Amp^r, P*trc* (*trc* promoter)-*pdxST*-rrnB cassette inserted into *Avr*II site of plasmid pETDuet-CadA	This work
pET-cadA-BADST	ColE1 *ori*, *lac*I gene, Amp^r, P_{BAD}-controlled *pdxST* and P_{T7}-controlled *cadA*	This work
pCWJ-pdxST	RSF *ori*, *lac*I gene, Cm^r, P_{trc}-controlled *pdxST*	This work

Table S2. Primers used in this work (restriction sites are underlined)

Primer	Sequence
CadA-NdeI-Duet-F	GGAATTCCATATGAACGTTATTGCAATATTG
CadA-KpnI-Duet-R	GGGGTACCTTATTTTTTGCTTTCTTCTTTC
PdxST-NcoI-F	CATGCCATGGCTCAAACAGGTACTGAACG
PdxST-SalI-R	ACGCGTCGACTTATACAAGTGCCTTTTGCTTATATTCCTCAACC
Trc-promoter-XbaI	CTAGTCTAGATTGACAATTAATCATCCGGCTCG
Trc-termi-XbaI	CTAGTCTAGAATTTGTCCTACTCAGGAGAGCGTTC
Para-Spe-Xba-F	CCCACTAGTCTAGAATCGATTTATTATGACAACTTGACGGCTACATCATTCAC
Para-Nco-Bam-R	CATAGGATCCATGGTTTATAACCTCCTTAGAGCTCGAATTCCC
